# Cellulose Plant-Derived Scaffolds as a Tool for Myometrium Modeling

**DOI:** 10.3390/ijms262210995

**Published:** 2025-11-13

**Authors:** Anastasiia V. Sokolova, Ivan K. Kuneev, Yuliya A. Nashchekina, Alisa P. Domnina

**Affiliations:** Institute of Cytology, Russian Academy of Sciences, 194064 Saint-Petersburg, Russia; kynejev@gmail.com (I.K.K.); aldomnina@mail.ru (A.P.D.)

**Keywords:** 3D culture, plant-derived, cellulose scaffold myometrium, smooth muscle cell, multilayered system

## Abstract

The myometrium is the smooth muscle layer of the uterus, whose dysfunctions are involved in various pathologies leading to infertility, such as adenomyosis and uterine fibroids. Developing relevant in vitro models of the myometrium is crucial for investigating the pathogenesis of these diseases. In this study, we propose a novel approach for cultivating mouse myometrial smooth muscle cells (SMCs) using plant-derived cellulose scaffolds. The scaffolds were obtained through the decellularization of green onion leaf, celery stalk, or bluegrass leaf, subsequently coated with collagen type I. We found that the structure of the green onion leaf scaffold provides unidirectional orientation of cultured cells, mimicking the tissue-specific organization of mouse myometrial SMCs in vivo. The mouse myometrial SMCs, cultured on this scaffold, proliferated, maintained viability up to 2.5 months, and retained the expression of the main markers of smooth muscle contractility (α-smooth muscle actin, transgelin, calponin, smooth muscle myosin heavy chains, connexin-43). To reproduce the native myometrium structure, a multilayered cultivation system was created. In a system of two overlaying scaffolds, cells also retained the viability and expression of smooth muscle contractility markers. The developed approach can be used for three-dimensional myometrium modeling to study the pathogenesis of myometrium-associated diseases.

## 1. Introduction

A current trend in cell biology is the shift toward studying three-dimensional (3D) cell models, which more accurately replicate the architecture and function of native human tissues [1,2,3]. The primary aim of most of these efforts is to create a cell culture model so closely approximating the native tissue that the need for using whole model organisms is minimized [4]. For human studies, such models are a means of overcoming the “valley of death” between preclinical and clinical research, making their development particularly crucial [5]. More accurate and complex in vitro models are essential for improving the reproducibility, effectiveness, and productivity of biological studies, leading to more predictive and human-relevant data that can be used for drug discovery and development [6].

3D cultures are multicomponent constructs, typically containing cells and scaffolds that replicate the extracellular matrix of tissues. They aim to simulate the native cellular microenvironment, allowing cells to attach, migrate, and differentiate. The main function of the scaffold is to provide a framework with suitable structure, stiffness, and sufficient adhesive properties for the cells used in the specific model [7,8]. The popularity of cellulose, a natural polymer based on D-glucose, in the creation of 3D scaffolds is increasing because it is a biocompatible and biologically inert material with mechanical strength and elasticity [9,10]. It can be obtained from various sources (e.g., synthesized by bacteria or obtained through the decellularization of plant tissues) and inexpensive to produce, while also offering diverse structures and shapes [9,10,11,12]. Cellulose is already actively used in fields such as water purification and drug delivery, but research on its application as a scaffold for tissue-engineered constructs has emerged only recently. To date, cellulose has been used for the regeneration of bone and cartilage tissue [13] and the creation of tissue-engineered constructs with vascular networks [14], as well as for in vitro modeling of striated muscle tissue [15,16].

3D cell cultures can serve as relevant models for studying disease pathogenesis and developing personalized treatments [17,18]. They are in demand across various medical fields, including reproductive medicine and gynecology [19,20]. Along with infertility caused by ovarian or endometrial pathologies, the failure to conceive or pregnancy loss at different stages can be caused by myometrial pathologies [21,22,23]. The myometrium is the smooth muscle layer of the uterus that generates the contractions necessary for processes like menstruation and labor. Aberrant (improper) myometrial contractions lead to preterm birth, insufficient labor progression, dysfunctional labor, and other issues that cause maternal or fetal complications and can result in severe disability and death. Conversely, the absence of myometrial contraction after delivery can result in postpartum hemorrhage, rapid loss of a large volume of blood, and maternal death [24,25]. The restoration of myometrial function is particularly urgent due to the modern shift in obstetric strategy, which involves a significant increase in the frequency of abdominal delivery by C-section [26]. Besides anomalies of contractile function and regeneration of the myometrium itself, other pathologies associated with this tissue lead to infertility and reduced quality of life (adenomyosis, uterine fibroids) [27,28,29,30]. The pathogenesis of such diseases is still not fully understood. Studying myometrial pathologies is difficult, and often impossible, because the risk to the woman, and during pregnancy, to the fetus, outweighs the potential benefit [31]. In this regard, it is essential to develop models that allow the study of disease pathogenesis and the advancement of treatment methods.

Traditionally, the most common model systems for investigating the mechanisms underlying myometrial pathological conditions have been animal organs and human myometrial strips, which have limitations primarily related to relevance and scalability, respectively [24,32,33]. Myometrial smooth muscle cells (SMCs) have also been isolated from patient biopsies and cultured in vitro as a more controlled experimental system [34]. Most in vitro cell culture studies involve growing cells on two-dimensional (2D) substrates. However, these conditions fail to reproduce the properties of native tissue. Unlike monolayer cultures, in 3D cell cultures, in which cells are placed on scaffolds mimicking the tissue architecture, a favorable environment is created to maintain intracellular processes and intercellular communication in a state closer to what we see in vivo [35,36,37]. Furthermore, 3D matrices can ensure the alignment and directional orientation of cells, which play critical roles in tissue functionality, especially muscle [38]. Thus, creating an in vitro 3D myometrium model is essential for fundamental research aimed at studying the mechanisms of uterine function and for medical purposes—developing personalized therapeutic approaches for diseases and drug testing.

Cellulose scaffolds of plant origin can be easily produced, are inexpensive to manufacture, have different architectures, are stable in aqueous solutions, and are suitable for in vitro 3D myometrium modeling. In this study, we developed a novel approach for 3D myometrium modeling, based on culturing mouse myometrial SMCs on a plant-derived collagen type I-coated cellulose scaffold.

## 2. Results

### 2.1. Plant-Derived Cellulose Scaffold for Myometrium Modeling In Vitro

**Figure 1:**
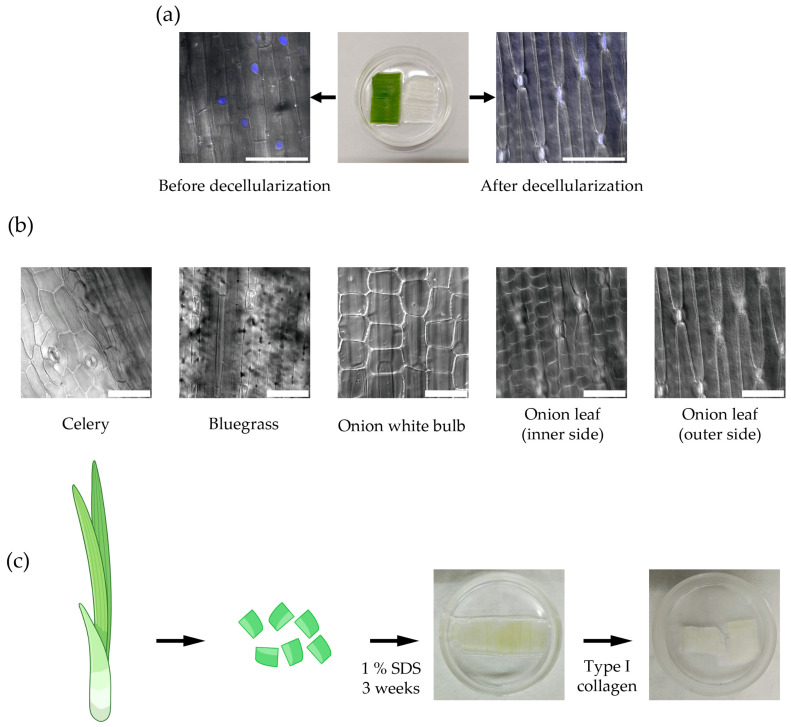
Plant-derived cellulose scaffold production. (**a**) The plant-derived cellulose scaffold before decellularization (left) and after decellularization (right). The effectiveness of the decellularization was confirmed by the absence of plant cell nuclei stained with DAPI (blue, confocal microscopy), scale bar 100 μm; (**b**) The structure of cellulose scaffolds obtained from various parts of different plants: celery stalk, bluegrass leaf, and green onion (confocal microscopy), scale bar 100 μm; (**c**) The scheme of producing cellulose scaffolds suitable for cell culturing (Figure partially created in BioRender. Domnina, A. (2025) https://BioRender.com/q79g5gc, accessed on 11 November 2025).

To reconstruct the architecture of the myometrium, we selected the appropriate parts of plants with a suitable structure that would promote the unidirectional alignment of SMCs. To remove plant cell components and obtain purified cellulose scaffolds, we used decellularization with 1% sodium dodecyl sulfate (SDS), as described by Cheng et al. [15]. Decellularization removes all the components of plant cells except for the cell wall, which is primarily composed of cellulose. The effectiveness of the decellularization protocol was confirmed by the absence of plant cell nuclei in the obtained scaffolds (Figure 1a).

We analyzed the structure of cellulose scaffolds obtained from various parts of several plant species, namely green onion leaves, celery stalks, and bluegrass leaves, using confocal microscopy (Figure 1b). It turned out that the scaffolds from the celery stalks, as well as the white parts of the green onion and the inner side of the green onion leaf, lack the structural features required for the aligned arrangement of myometrium cells (long, extended channels of shallow depth) (Figure 1b). In contrast, bluegrass leaf scaffolds feature deep channels that may prevent the formation of a continuous cell layer. However, scaffolds made from the outer side of the green onion leaf have shallow channels elongated in one direction, making this scaffold most promising for myometrium model development.

Our previous research found that cells do not attach to untreated plant-derived cellulose scaffold [39]. Thus, to promote cell adhesion all of the plant-derived cellulose scaffolds were treated with collagen type I according to a previously developed protocol [39]. Figure 1c illustrates the method for producing cellulose scaffolds from a green onion leaf suitable for cell culturing.

### 2.2. Isolating and Maintaining Primary Mouse Myometrial Smooth Muscle Cells

**Figure 2:**
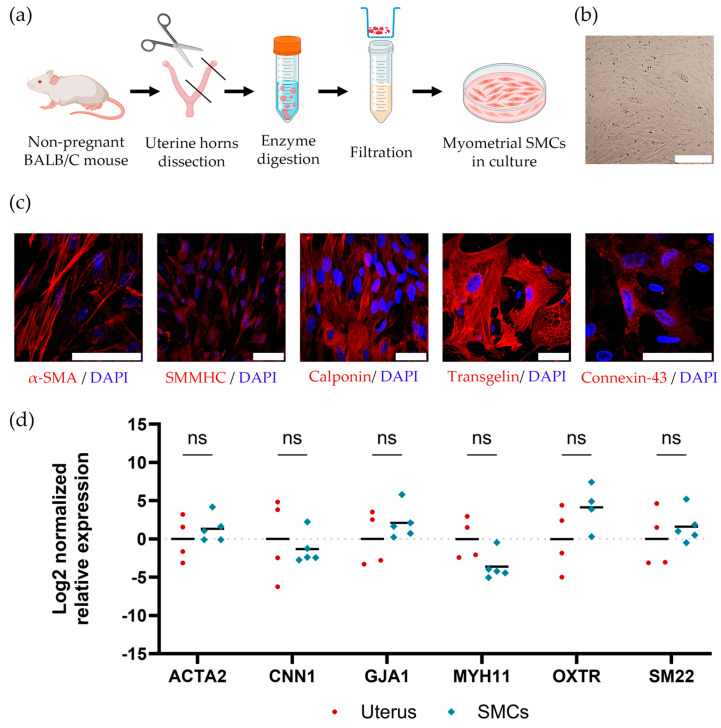
(**a**) The scheme of the isolation of mouse myometrial SMCs (Figure partially created in BioRender. Domnina, A. (2025) https://BioRender.com/q79g5gc, accessed on 11 November 2025); (**b**) The fibroblast-like morphology of isolated mouse myometrial SMCs, scale bar 300 μm (**c**) The immunocytochemical staining of smooth muscle markers: α-SMA, SMMHC, calponin, transgelin, and connexin-43 in mouse myometrial SMCs (secondary antibody conjugated with Cy3 (red), nucleus stained DAPI (blue), scale bar 50 μm; (**d**) The expression of smooth muscle marker genes and oxytocin receptor gene in native mouse uterus and mouse myometrial SMCs, RT-PCR. Data are presented as geometric mean (GM, bars) and individual values (*n* ≥ 3) (normalized to the Uterus control). Statistical significance was determined using the Mann–Whitney U test; ns indicates *p* > 0.05.

Mouse SMCs were obtained from the myometrium of non-pregnant female BALB/c mice. The process for the isolation of mouse myometrial SMCs is presented in Figure 2a.

Isolated cells represented a population of fibroblast-like cells (Figure 2b), and these cells demonstrated the expression of smooth muscle markers, such as α-smooth muscle actin (α-SMA), smooth muscle myosin heavy chains (SMMHC), calponin, transgelin, and connexin-43, as visualized by immunocytochemistry (Figure 2c). To compare the expression of smooth muscle marker genes in native mouse uterus and mouse myometrial SMCs, we used the RT-PCR method. We also identified oxytocin receptor gene expression, which is important in myometrial contractile function (Figure 2d). We found that the gene expression levels in mouse myometrial SMCs are comparable to the levels in native tissue, which confirms the contractile phenotype of the obtained primary SMCs.

### 2.3. Cultivating Mouse SMCs on the Plant-Derived Cellulose Scaffolds

**Figure 3:**
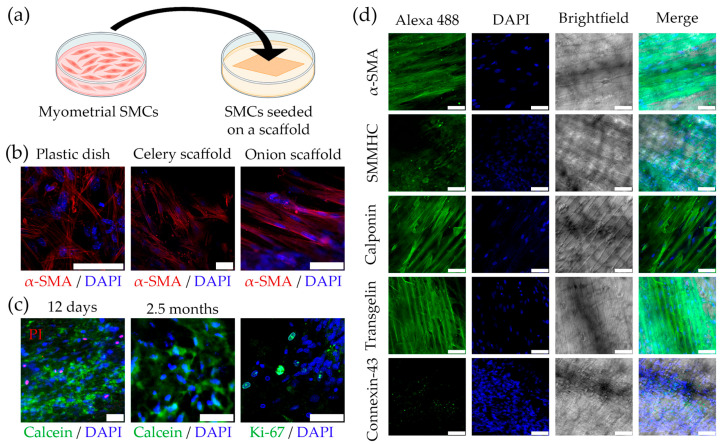
Cultivation of mouse SMCs on the plant-derived cellulose scaffolds. (**a**) The scheme of the experiments (Figure partially created in BioRender. Domnina, A. (2025) https://BioRender.com/q79g5gc, accessed on 11 November 2025); (**b**) The immunocytochemical staining of smooth muscle markers α-SMA (red) in SMCs cultured on different scaffolds. Random orientation of SMCs is obviously seen on a plastic culture dish and on the celery scaffold, while unidirectional SMCs alignment is typical for the scaffold from the outer side of the scaffold from the green onion leaf, scale bar 50 μm; (**c**) The SMCs cultured on green onion leaf for 12 days and 2.5 months scaffold stained with PI (red), DAPI (blue) and Calcein (green). It is seen a small number of PI-stained cell nuclei (dead cells) and a large number of live cells stained with Calcein, which indicates the maintenance of SMCs viability during cultivation for up to 2.5 months. Immunocytochemical staining for the proliferating cell marker Ki-67 (green) indicated proliferation of the SMCs when cultured on the scaffold, scale bar 50 μm; (**d**) The immunocytochemical staining of SMCs cultured on green onion leaf for smooth muscle markers (α-SMA, SMMHC, transgelin, calponin, and connexin-43), scale bar 50 μm.

The mouse myometrial SMCs were seeded onto collagen-coated cellulose scaffolds and analyzed after 12 days of cultivation. The method of the experiment is illustrated in Figure 3a.

We observed that mouse myometrial SMCs cultivated on plant-derived scaffolds successfully attached and distributed on the scaffold. In addition, we found that the spreading and orientation of the cells depended on the scaffold structure. On the celery scaffold, the cells were oriented randomly, as on a standard culture dish (Figure 3b). Thus, this scaffold does not provide the necessary structure for the unidirectional alignment of the cells, which is one of our main requirements for modeling the myometrium. The bluegrass leaf scaffold features deep channels that prevent the formation of a continuous layer of SMCs aligned in one direction, which is crucial for the myometrium. Only when the cells were cultured on the outer side of the scaffold from the green onion leaf, we were able to achieve unidirectional cell alignment (Figure 3b). This scaffold was not only the most flexible and soft but also elastic and durable enough to maintain its shape and cell growth. Additionally, it was convenient for manipulation during cultivation. Thus, the collagen-coated cellulose scaffold from the outer side of the green onion leaf was the most suitable for myometrium modeling. Analysis of mouse myometrial SMCs seeded on this scaffold revealed a small number of Propidium Iodide (PI)-stained cell nuclei (dead cells) and many live cells stained with Calcein, which indicates the maintained viability of the SMCs during cultivation for up to 2.5 months (Figure 3c). Immunocytochemical staining for the proliferating cell marker Ki-67 indicated SMC proliferation when cultured on the scaffold (Figure 3c). The SMCs also exhibited specific staining of intracellular components for smooth muscle markers (α-SMA, SMMHC, transgelin, calponin, and connexin-43), which confirms the maintenance of the expression of these smooth muscle markers in the cells on the scaffold (Figure 3d).

### 2.4. Developing a Multilayer Model of Myometrium Based on Plant-Derived Cellulose Scaffolds

**Figure 4:**
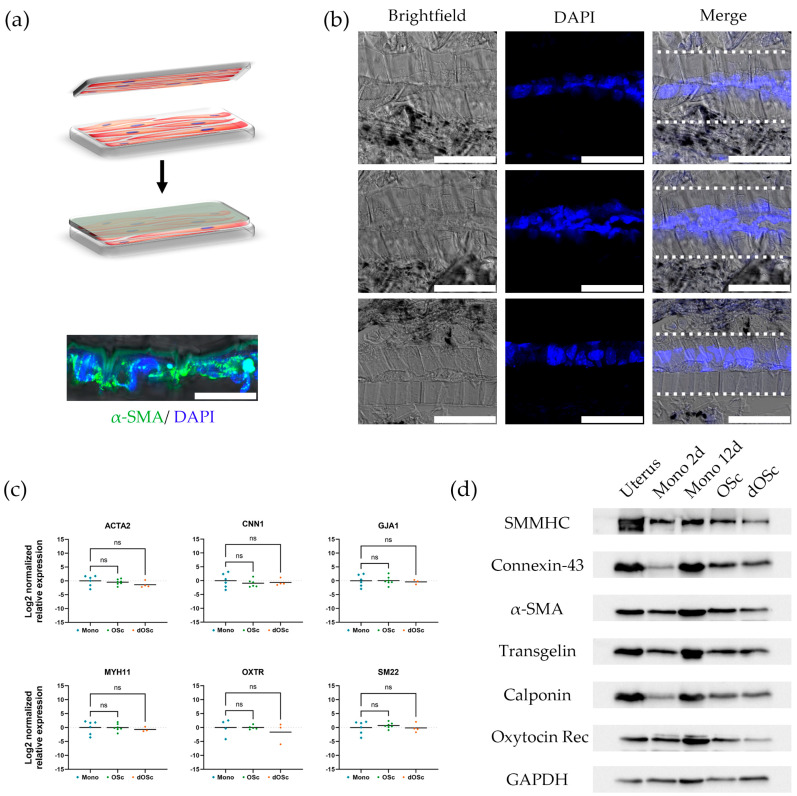
Development of a multilayer model of myometrium. (**a**) The scheme of the obtaining the multilayer structure using two green onion scaffolds with SMCs placed one on top of the other such that the cell layers faced each other (Figure partially created in BioRender. Domnina, A. (2025) https://BioRender.com/q79g5gc), the immunocytochemical staining of the multilayer construction for α-SMA (green), scale bar 50 μm; (**b**) The histological observation of cross-section of the multilayer construction, nuclei are stained with DAPI (blue), superimposed layers are marked with dotted lines, scale bar 50 μm; (**c**) The RT-PCR analysis of expression of key contractile smooth muscle marker genes in primary mouse myometrial SMCs cultured on a plastic dish, on a green onion leaf cellulose scaffold, and in the multilayer construct. Geometric mean (GM, bars) and individual values (*n* ≥ 3), normalized to the control (Monolayer culture), are shown. Statistical significance was determined using the Kruskal–Wallis H test with Dunn’s post hoc test for pairwise comparisons versus the control group; ns indicates *p* > 0.05; (**d**) The Western blot analysis of key contractile smooth muscle marker proteins in primary mouse myometrial SMCs cultured on a plastic dish, on a green onion leaf cellulose scaffold, and in the multilayer construct.

The myometrium is the multilayer muscular wall of the uterus. Thus, to mimic the native structure of the myometrium, we developed a multilayer structure using two green onion scaffolds with SMCs placed one on top of the other, such that the cell layers faced each other (Figure 4a).

SMCs were cultured in this way for 7 days and subsequently analyzed. A histological analysis of cross-sections was performed to characterize the location of the cells in the structures and the internal architecture of the obtained multilayer constructs (Figure 4b). We found that the cells between the layers retained unidirectional alignment and viability. We compared the expression of key contractile smooth muscle marker genes in primary mouse myometrial SMCs cultured on a plastic dish, on a green onion leaf cellulose scaffold, and in a multilayer construct using the RT-PCR method (Figure 4c). We found similar levels of gene expression in all the samples. We also confirmed the presence of the corresponding proteins in all the samples by Western blotting analysis (Figure 4d).

Altogether, our results provide proof-of-concept for creating a relevant multilayer model of the myometrium based on cellulose scaffolds from a green onion leaf.

## 3. Discussion

In this work, we present a novel approach for 3D myometrium modeling based on a plant-derived cellulose scaffold. To date, the most commonly used models for studying the mechanisms underlying myometrial pathologies are animal models and human myometrial strips [24]. Besides that, myometrial SMCs have been isolated from patient biopsies and cultured in vitro. However, the serious limitation of the animal models (usually mice) is a significant difference in the structure of the uterus, particularly the myometrium, between rodents and humans [24]. As for the use of SMCs, these cells, under traditional 2D culturing conditions, can switch between a differentiated contractile phenotype and a dedifferentiated “synthetic” phenotype. The latter is characterized by a loss of SMC marker gene expression, increased synthesis of extracellular matrix (ECM) components, and enhanced proliferative and migratory SMC activity when exposed to various environmental stimuli and signals [40,41]. Eventually, these phenotypic changes lead to a loss of cell tissue-specificity [42].

In contrast to monolayer cultures, 3D cells cultures, in which cells are placed on scaffolds mimicking tissue architecture and containing natural ECM proteins, create a favorable environment for maintaining normal intracellular functions and interactions resembling native conditions [35,36,37,43]. The 3D matrix also provides cells with a milieu containing growth factors and cytokines that would otherwise be lost in the culture medium [35,44]. Such cultures also provide mechanical cues transmitted from the surrounding ECM to the cells via integrins, which are transmembrane receptors, to the cytoskeletal network, thereby influencing intracellular processes [45,46].

Relatively few studies on the development of 3D myometrium are available in the literature. Young and colleagues cultured human myometrial SMCs using polyglactin 910 (Vicryl) meshes [47]. In another study, researchers used magnetic 3D bioprinting to assemble human myometrial cells into rings, which were then used to investigate uterine contractility under the influence of various inhibitors [38]. There are also reports of culturing immortalized human myometrial cells incorporated into a gel made of type I collagen [48]. We believe that, despite the advanced properties of the above-mentioned myometrium cultures, insufficient attention is focused on the directional growth of the cells. In the native myometrium, muscle cells form multilayered structures with the specific orientation necessary to generate the required tension [34,38]. The primary function of the myometrium is contractile, and uterine contraction is a 3D-coordinated process that should be studied in a relevant 3D system.

In the 3D myometrium model we developed, decellularized plant tissues were used as scaffolds for cell growth. Due to their architecture, they allow for controlled cell alignment in vitro, unlike most other substrates. In recent years, scaffolds made from decellularized plant parts have gained popularity in regenerative medicine [49,50,51,52,53]. The plant ECM mainly consists of cellulose, typically containing pectin and hemicellulose. Cellulose, the most abundant organic polymer on Earth, has been found in numerous studies to be cytocompatible [54]. The abundance of plants makes their decellularized structures a stably available, ethically sourced, and inexpensive alternative to decellularized animal and human tissues and organs for creating tissue-engineered constructs.

Primarily for this reason, we chose decellularized plant parts as the basis for scaffolds for culturing myometrial SMCs. First, we focused on selecting an adequate matrix—identifying a plant material with architecture suitable for myometrial SMC culture and optimizing the decellularization conditions. In the functioning of both skeletal and smooth muscle tissue, the orientation of the muscle cells plays a crucial role. Therefore, in addition to meeting the basic scaffold requirements (mechanical strength, physicochemical stability, biocompatibility, and biological inertness), the matrix for SMC culture must ensure the correct orientation of the muscle cells. Since the ECM structure is preserved during decellularization, a matrix suitable for the chosen cell type can be selected beforehand. In the current work, the architecture of various plant materials was analyzed, and their accessibility and ease of preparation were evaluated. As a result, several candidates were selected for the cellulose scaffold: celery stalks, green onion leaves, and bluegrass leaves. The cellulose scaffolds were obtained through decellularization of the selected plants, and to ensure cell adhesion, the resulting scaffolds were coated with type I collagen using a previously developed method [39].

There are published data on using decellularized fruits and vegetables, such as carrot, broccoli, cucumber, potato, apple, asparagus, green onion, leek, and celery, as scaffolds for the differentiation of muscle cells into striated muscle fibers [15]. However, our work investigating scaffolds from green onion, celery, and bluegrass as potential candidates indicates that the outer side of the green onion leaf has the most suitable structure for myometrial cell culture, as it features unidirectional channels of shallow depth. This result aligns with the success of studies on skeletal muscle cell culture on various plant scaffolds [14]. Mechanical properties of cellulose scaffolds are widely described in the literature [15,50,51].

Mouse myometrial SMCs were chosen to assess the feasibility of culturing SMCs on a plant-derived scaffold. Cells were isolated from the uterus of non-pregnant BALB/c mice and characterized. The isolated cells presented a fibroblast-like structure and expressed smooth muscle markers, such as α-SMA, SMMHC, transgelin, calponin, connexin-43, and oxytocin receptor, confirming the smooth muscle phenotype. In our previous studies we found that cells do not attach to untreated plant-derived cellulose scaffold [39]. Therefore, the scaffolds were treated with type I to promote cell adhesion. Cell retention on the scaffold surface was observed. The analysis of cell distribution across the scaffolds confirmed that the outer side of the green onion leaf possessed the most suitable structure for SMC culture. Its architecture, featuring elongated channels, allows the cellulose scaffold to facilitate cell alignment in one direction—which is critically important for uterine SMCs—while the scaffold’s pores are not so deep as to prevent the formation of a unified muscle cell layer.

Further analysis revealed that the cells on the cellulose scaffold maintained their viability and proliferated, confirming the potential for using plant-derived cellulose scaffolds as a substrate for myometrial SMC culture. A key feature of SMC culture on the green onion leaf scaffold was the unidirectional alignment of the cells, unlike cells cultured on plastic. Moreover, SMCs cultured on the green onion scaffold maintained the expression of smooth muscle markers. Thus, cells can be cultured on this substrate while preserving their smooth muscle phenotype, viability, and proliferative capacity, and the scaffold provides a unidirectional cell orientation, which is crucial for the myometrium. Furthermore, the viability of the cells on the green onion scaffold was maintained for 2.5 months, which confirms the cytocompatibility of the resulting scaffold and the possibility of using the cellulose scaffold-SMC construct for a long duration, a particularly valuable feature when working with human material.

The myometrium is a multilayered structure, so the next goal was to join several cell-seeded cellulose scaffolds to create and investigate a single multilayer construct. For this, several single-layer green onion scaffolds with SMCs were combined by stacking the constructs, such that the cell layers faced each other and were joined in different directions. The retention of SMCs within the multilayer construct was demonstrated, as was their expression of smooth muscle markers α-SMA, SMMHC, transgelin, calponin, connexin-43, and oxytocin receptor. Furthermore, the formation of multilayer sheets from the stacked SMCs was observed. This suggests the fundamental possibility of creating a multilayer construct based on the green onion leaf cellulose matrix and SMCs to study myometrial properties in a multilayer system, as occurs in native tissue.

Thus, the green onion leaf scaffold with myometrial SMCs constitutes a stable 3D construct containing cells that retain the smooth muscle phenotype. This construct is easy to manipulate because the scaffold is sufficiently durable and flexible. Moreover, the confirmation of SMC retention upon stacking multiple cell layers engenders the creation of a multilayer model in which SMCs maintain their smooth muscle phenotype and the orientation characteristic of muscle cells in native tissue. The future also holds the possibility of creating a multilayer construct with different cell types to model all layers of the uterine wall. It is anticipated that similar constructs could be used to develop a 3D myometrium model based on human cells for a personalized approach to the diagnosis and treatment of myometrial diseases.

In summary, the developed 3D culture system can serve as an in vitro myometrium model for fundamental research aimed at studying uterine function and for medical purposes, including the development of personalized approaches to therapy for myometrium-related diseases and drug testing. The described model is a preclinical mouse model, and the applicability to human cells needs to be verified in the future. The use of 3D myometrial cell culture brings us closer to creating a 3D myometrium model capable of more fully reflecting tissue properties, which will allow more extensive in vitro studies of myometrial pathologies. We hope that the application of this and similar model systems will facilitate the development of personalized approaches to the treatment of gynecological diseases, thereby avoiding the negative consequences of inappropriate treatment, reducing the economic costs of subsequent complication correction, and improving patients’ quality of life.

## 4. Materials and Methods

### 4.1. Preparation of Cellulose Scaffolds

Cellulose scaffolds were made from green onion leaf, celery stalk, or bluegrass leaf. Green onion and celery were obtained from local market and stored at 4 °C. Bluegrass was grown up from seeds obtained from garden market. The plant part designated for scaffold creation (the stalk of celery, the leaf of onion, or the leaf of bluegrass) were isolated and cut into small pieces (1 × 1–3 cm). Next, each excised plant fragment was decellularized by detergent treatment for 3 weeks under continuous agitation of the 5 mL tube containing the precursors on an orbital shaker at 30 rpm at room temperature in 5 mL of 1% SDS solution (AppliChem GmbH, Darmstadt, Germany) that was replaced weekly. (the protocol adopted from [15]). The resulting scaffolds were then purified to remove detergent. The detergent was removed by washing the samples in 5 mL of phosphate-buffered saline (PBS) (Biolot, Saint-Petersburg, Russia) for 48 h on an orbital shaker at 30 rpm with the PBS solution replaced at least five times. After that, the scaffolds were sterilized by treatment with 5 mL of 70% (*v*/*v*) solution of ethyl alcohol for 1 h under slow rocking of the samples on a rocker shaker. Washing of the cellulose scaffolds from ethanol was carried out by intensive pipetting in 2 mL of PBS with ten buffer changes. Following washing, the scaffolds were transferred to a new sterile tube where they were stored for a maximum of one month in sterile PBS until the collagenization. To improve the adhesion properties of the cell attachment surface, the scaffolds were treated with collagen Type I according to a previously developed procedure [38]. The cellulose scaffolds were coated with a collagen Type I solution (at a concentration of 2 mg/mL) in the presence of 0.1% acetic acid, and incubated at a temperature of −20 °C for 24 h. The concentration of 2 mg/mL for collagen Type I was selected as the standard working concentration for scaffold surface coating based on established protocols in tissue engineering [39,55,56]. Subsequently, the scaffolds were freeze-dried at a temperature of −50 °C and a pressure of 0.1 mm Hg for 8 h. The obtained collagenized scaffolds were stored at a temperature of −20 °C for no more than a month. Prior to use, the scaffolds were sterilized by ozone treatment, then washed with sterile PBS at least three times. To evaluate the efficiency of cell structure removal after decellularization, the presence of plant cell nucleus residues in the obtained scaffolds was determined by staining with the fluorescent intercalating dye DAPI (Sigma-Aldrich, Saint Louis, MO, USA). Imaging was conducted using an Olympus FV3000 laser scanning confocal microscope (Olympus, Tokyo, Japan). Decellularization efficiency was confirmed by the absence of plant cell nuclei in the scaffold structure.

### 4.2. Isolating and Cultivating of Mouse Myometrial SMCs

For myometrial SMCs isolation and uterine tissues samples obtaining 30 non-pregnant female Balb/C mice 8 weeks old were used. The animals were purchase from Federal State Unitary Enterprise “Nursery of Laboratory Animals “Rappolovo”, Russia. Mice were housed in animal facility of the Institute of Cytology of the Russian Academy of Sciences under standard conditions, with ad libitum access to food pellets and tap water. The work with laboratory animals was carried out according to ethical principles and all experiments were approved by the Bioethics Commission of the Institute of Cytology of the Russian Academy of Sciences (protocol No. 03/24 dated 6 June 2024). Mice were euthanized by cervical dislocation under ether anesthesia. Murine myometrial SMCs were isolated under sterile conditions according to a published protocol [57]. The uteri were isolated from the mice, and the uterine horns were cut longitudinally and washed with Hanks’ balanced saline solution (HBSS). A subset of the resulting uterine tissue pieces was frozen in liquid nitrogen and used as a native tissue control for subsequent gene and protein expression analysis (RT-PCR and Western blotting). After washing with HBSS, the prepared uterine horns were placed in sterile centrifuge tubes and exposed to a 2.5 mg/mL solution of dispase (Worthington Bioch. Corp., Lakewood, NJ, USA) in HBSS for 1 h at 4 °C, followed by 1 h at room temperature and 35 min at 37 °C. Then, after three HBSS washes, the uterine horns were incubated in a 0.15 mg/mL collagenase I solution (Gibco, **Grand Island, NY,** USA) in HBSS. Following incubation, the tubes were shaken manually, and the tissue fragments were washed three times with HBSS, and subsequently treated with a solution of 0.25% trypsin (Gibco, USA) and 0.5% collagenase I in HBSS for 1 h at 37 °C. The resulting cell suspension was then passed through a 40 μm filter. The filtered cell suspension was centrifuged at 400 g for 5 min. After removal of the supernatant, the cells were resuspended in DMEM/F12 culture medium (Gibco, USA) containing 10% fetal bovine serum (FBS) (HyClone, Logan, UT, USA), 1% penicillin/streptomycin mixture (Gibco, USA), and 1% glutamine (Gibco, USA) and cultured at 37 °C and 5% CO_2_ for 2 days. The cells were then transferred to a new culture vessel or seeded onto the scaffolds and cultured under the same conditions for 12 days with complete culture medium replacement every 3 days. For individual experiments, cell nuclei were further stained with Hoechst 33342 (Sigma-Aldrich, Saint Louis, MO, USA), a dye that intercalates in live cell DNA, according to the manufacturer’s protocol.

### 4.3. Seeding of Murine Myometrial SMCs onto Cellulose Scaffolds

The cell seeding was performed using SMCs isolated from mouse myometrium according to the protocol described above, at passage 1. To settle into the scaffolds, the cells were detached from the substrate on the second day after isolation using a 0.05% trypsin and EDTA solution, 5 × 10^5^ cells were used to seed 1 scaffold with an area of 2 cm^2^. The SMCs were centrifuged at 400× *g* for 5 min and resuspended in 40 μL of culture medium. The prepared scaffold was placed on a dry, non-adhesive culture dish with a diameter of 3.5 cm, and the cell suspension was uniformly applied onto the scaffold. The resulting construct was then incubated for 30 min at 37 °C and 5% CO_2_. Subsequently, 2 mL of the culture medium was added, and the cells were cultured on a scaffold for 12 days under the same conditions, with a complete medium change every 3 days. Assessment of the viability of the SMCs during cultivation on plastic substrates and in a three-dimensional scaffold structure was performed by confocal microscopy. To evaluate viability, the cultured cells were stained with the fluorescent dyes PI (Sigma, USA) (50 μg/mL, incubated for 20 min in the dark at 37 °C), which stains the nuclei of dead cells, and Calcein (Servicebio, Wuhan, China), which stains the cytoplasm of living cells, according to the manufacturer’s protocol. The samples were subsequently analyzed by fluorescence microscopy using a NIB-620FL microscope (Nexcope, Ningbo, China).

### 4.4. Developing of a Multilayer Scaffold-Cell Construct

Pairs of green onion scaffolds seeded with SMCs, prepared according to the above-described protocol, were combined 7 days after the formation of the constructs. The combination was performed by superimposing one construct onto another so that the cell layers were facing each other and connected in different directions. The resulting multilayer constructs were cultured under the same conditions as described above for an additional 7 days, followed by analysis. To visualize the arrangement of the cells and the internal architecture of the resulting multilayer constructs, they were frozen in liquid nitrogen, and 10 μm-thick cross-sections were made using a Bright Co Ltd. cryotome (London, UK). The obtained sections were then examined by immunocytochemistry.

### 4.5. Immunocytochemical Staining

The SMCs cultured on plastic or scaffolds were washed three times with PBS for 5 min, fixed in 10% formalin for 15 min, and washed again three times with PBS for 10 min. The cells were then permeabilized with a 1% Triton X-100 solution for 30 min, followed by blocking of non-specific binding sites in PBS containing 1% BSA (Sigma, USA), 5% normal goat serum, and 0.3% Triton X-100 for 1 h. Incubation with the primary antibodies was carried out overnight at 4 °C. Primary rabbit anti-mouse antibodies (Abclonal, Wuhan, China) against the following antigens were used: α-smooth muscle actin (ACTA2) (mAb), smooth muscle myosin heavy chain (SMMHC) (mAb), calponin (CNN1) (mAb), transgelin (TAGLN) (pAb), oxytocin receptor (OXTR) (mAb), connexin 43 (GJA1) (mAb), and Ki-67 (Abcam, Cambridge, MA, USA). Antibodies were diluted 1:100 in PBS containing 1% BSA and 0.5% Triton X-100. Subsequently, the samples were washed three times with PBS containing 0.3% Triton X-100 for 10 min. The samples were then incubated with secondary goat anti-rabbit IgG antibodies conjugated to Alexa Fluor 488 (Invitrogen, South San Francisco, CA, USA) at a dilution of 1:1000 (in PBS containing 1% BSA and 0.3% Triton X-100) or with secondary goat anti-rabbit IgG antibodies conjugated to Cy3 (Abcam, UK) at a dilution of 1:100 (in PBS containing 1% BSA and 0.3% Triton X-100) for 45 min in the dark. Following incubation, the samples were washed three times with PBS for 10 min. Cell nuclei were visualized by applying 1 μg/mL of DAPI for 5 min. After staining, the scaffolds were placed in a 4-well cuvette with a borosilicate glass bottom and examined by confocal microscopy.

### 4.6. Western Blot Analysis

For the Western blot analysis, SMCs were lysed using a standard protocol [51] with lysis buffer containing 50 mM Tris-HCl (pH 7.5), 150 mM NaCl, 1 mM EDTA, 1 mM EGTA, 10% glycerol, 1% Triton X-100, 1 mM NaF, 0.5 mM PMSF, and protease and phosphatase inhibitor cocktail (ThermoScientific, Waltham, MA, USA). Whole uteri and scaffolds with cells were frozen in liquid nitrogen, triturated, then the lysing buffer was added, and the mixture was homogenized by pipetting, incubated for 30 min on ice, and then lysate was spinning the at 13,000× *g* for 30 min at 4 °C. Protein concentration was determined by the Bradford method using a GeneQuant 1300 spectrophotometer (GE Biochrom, Cambridge, UK). Electrophoretic protein separation (SDS-PAGE) was performed by the Laemmli method [58] using 5%, 10%, or 12% polyacrylamide gels. The proteins were then electrotransferred from the polyacrylamide gel to a nitrocellulose membrane (Amersham, Munich, Germany) according to the manufacturer’s protocols (Bio-Rad, Hercules, CA, USA). To visualize protein bands, the membrane was stained with Ponceau S dye (Sigma, USA). Next, the membrane, washed free of Ponceau S dye in PBS with 0.05% Tween 20, was placed in a blocking solution (5% skim milk (Bio-Rad, USA) in PBS with 0.05% Tween 20) overnight at 4 °C. The membrane was subsequently incubated in primary antibodies for 3 h at room temperature. Primary rabbit antibodies against α-SMA (1:5000), SMMHC/MYH11 (1:1000), calponin (1:5000), transgelin (1:1000), connexin 43 (1:1000), oxytocin receptor (1:1000) (Abclonal, China), and glyceraldehyde-3-phosphate dehydrogenase (GAPDH) (1:2000) (Cell Signaling, Danvers, MA, USA) were used. As secondary antibodies, goat anti-rabbit IgG immunoglobulin antibodies conjugated with horseradish peroxidase (Sigma, USA) (1:10,000) were used. Peroxidase activity was detected by an ECL kit (Millipore, Burlington, MA, USA); chemiluminescence was recorded in a ChemiDocTM gel documentation system (Bio-Rad, USA).

### 4.7. Gene Expression Analysis

#### 4.7.1. RNA Isolation

A cell suspension was prepared enzymatically from the monolayer culture prior to RNA isolation. Cells were washed from growth medium with an equal volume of PBS, then incubated with 0.05% trypsin solution containing EDTA and phenol red (Thermo Scientific, Waltham, MA, USA) until detached at 37 °C and 5% CO_2_. Trypsin was then inactivated by adding a 3-fold larger volume of culture medium containing 10% FBS, and the cells were pelleted by centrifugation with an acceleration of 200× *g* for 5 min. Total RNA was isolated on columns using the RNA Solo kit (Evrogen JSC, Moscow, Russia) according to the manufacturer’s protocol. The scaffolds populated with SMCs were pre-homogenized to a fine powder in liquid nitrogen with a ceramic mortar and pestle. Isolated RNA was stored in nuclease-free water (Evrogen, Russia) at −20 °C for a maximum of 6 months prior to use. The concentration and purity of the resulting RNA were determined using a NanoDrop One spectrophotometer (Thermo Scientific, USA). Samples with absorption ratios A260/A230 > 2.0 and A260/A280 > 1.9 were considered clean and usable. RNA integrity after isolation was checked by analytical gel electrophoresis using a gel containing 1.5% agarose (Sigma, USA) and 0.5 μg/mL ethidium bromide (Sigma, USA). BE-DNA tris-acetate buffer (Biolabmix, Novosibirsk, Russia) was used for gel preparation and electrophoresis.

#### 4.7.2. Reverse Transcription and Real-Time Quantitative PCR (RT-qPCR)

Reverse transcription was performed using the MMLV RT kit (Evrogen, Russia) with random decanucleotide primers according to the manufacturer’s protocol immediately prior to PCR. The reaction temperature conditions were: 45 min at 40 °C, 10 min at 70 °C, lid temperature = 95 °C.

RT-qPCR was performed using 5× qPCRmix-HS SYBR ready mix (Evrogen, Russia). Amplification and analysis were performed in a CFX96 Opus Real-Time PCR System (Bio-Rad, Hercules, CA, USA) running CFX Maestro 2.3 software (Bio-Rad, Hercules, CA, USA). The reaction conditions were: Initial denaturation: 95 °C for 3 min; Cycling (40 iterations): Denaturation: 95 °C for 20 s; Primer annealing: 60 °C for 20 s; Elongation: 72 °C for 30 s Melting curve analysis: Heating from 65 °C to 95 °C in increments of 0.5 °C every 5 s. The sequences of all primers used (Evrogen, Russia) are shown in Table 1. Relative gene expression values were calculated using the 2^−ΔΔCt^ method in Bio-Rad Maestro 2.3 software (Bio-Rad, USA). The final normalization and statistical analysis of the data were then performed using GraphPad Prism 10.2.1 (GraphPad, Boston, MA, USA).

## Figures and Tables

**Table 1:** Primer sequences for real-time PCR.

Target Name	Gene	Direction	Primer Sequence (5′-3′)
Mouse 18S	*18S*	Forward	GCAATTATTCCCCATGAACG
		Reverse	GGCCTCACTAAACCATCCAA
Mouse Connexin 43 (Gap junction alpha-1 protein (GJA1))	*GJA1*	Forward	GTGCCGGCTTCACTTTCA
		Reverse	GGAGTAGGCTTGGACCTTGTC
Mouse Oxytocin Receptor	*OXTR*	Forward	GTGCAGATGTGGAGCGTCT
		Reverse	GTTGAGGCTGGCCAAGAG
Mouse Alpha SMA-2	*ACTA2*	Forward	GTCCCAGACATCAGGGAGTAA
		Reverse	TCGGATACTTCAGCGTCAGGA
Mouse Calponin	*CNN1*	Forward	GGTGAAACCCCACGACATCTT
		Reverse	TTTGTCTTGGCCATGCTGG
Mouse Myosin heavy chain 11	*MYH11*	Forward	CATCCTGACCCCACGTATCAA
		Reverse	ATCGGAAAAGGCGCTCATAGG
Mouse Transgelin (TAGLN)	*SM22* alpha	Forward	CGATGGAAACTACCGTGGAGA
		Reverse	TGAAGGCCAATGACGTGCT

## Data Availability

The original contributions presented in this study are included in the article. Further inquiries can be directed to the corresponding author.

## Abbreviations

The following abbreviations are used in this manuscript:

3D	three-dimensional
SMCs	smooth muscle cells
SDS	sodium dodecyl sulfate
α-SMA	α-smooth muscle actin
SMMHC	smooth muscle myosin heavy chains
PI	Propidium Iodide
ECM	extracellular matrix
PBS	phosphate-buffered saline
DAPI	4′,6-diamidino-2-phenylindole
HBSS	Hanks balanced saline
FBS	fetal bovine serum
